# Ring Opening Reactions of β‐Propiolactam in Superacidic Media

**DOI:** 10.1002/chem.202104086

**Published:** 2022-01-05

**Authors:** Stefanie Beck, Vanessa Rück, Lea‐Viktoria Pietsch, Christoph Jessen, Andreas J. Kornath

**Affiliations:** ^1^ Department of Chemistry Ludwig-Maximilian University of Munich Butenandtstr. 5–13 81377 München Germany; ^2^ F-Select GmbH Semmelweisstraße 5 82152 Planegg Germany

**Keywords:** beta-propiolactam, natural bond orbital calculations, ring opening, superacidic media, thionylimide moiety

## Abstract

The reaction of β‐propiolactam in the superacidic systems HF/*M*F_5_ (*M*=Sb, As) led to the formation of monoprotonated 3‐aminopropanoyl fluoride in the form of [C(O)F(CH_2_)_2_NH_3_][SbF_6_] and [C(O)F(CH_2_)_2_NH_3_][AsF_6_]. In the presence of traces of water, the diprotonated species β‐alanine [C(OH)_2_(CH_2_)_2_NH_3_][AsF_6_]_2_ was synthesized for the first time. All salts were characterized by low‐temperature infrared and Raman spectroscopy. Additionally, single‐crystal X‐ray analyses were conducted in the case of [C(O)F(CH_2_)_2_NH_3_][SbF_6_] and [C(OH)_2_(CH_2_)_2_NH_3_][AsF_6_]_2_. By using SO_2_ instead of HF as the solvent, the salt [C(OH)_2_(CH_2_)_2_NHSO][SbF_6_]_2_ was obtained, and single‐crystal X‐ray analysis of this salt containing a thionylimide moiety was conducted. For the formation of these open‐chain compounds, an acyl cationic species as intermediate is assumed, which is formed from N‐protonated β‐propiolactam. Quantum chemical calculations at the B3LYP/aug‐cc‐pVTZ and MP2/aug‐cc‐pVTZ levels of theory were carried out to gain a better understanding of the formation and the structural properties of protonated β‐propiolactam.

## Graphical abstract

**Opening up possibilities**: The reaction of β‐propiolactam in the superacidic systems HF/*M*F_5_ and SO_2_/HF/*M*F_5_ (*M*=Sb, As) leads exclusively to previously unknown open‐chain compounds. With HF as the solvent, salts of monoprotonated 3‐aminopropanoyl fluoride and diprotonated β‐alanine are obtained, depending on the reaction conditions. Interestingly, the change of the solvent to SO_2_ leads to the formation of a salt containing a thionylimide moiety.

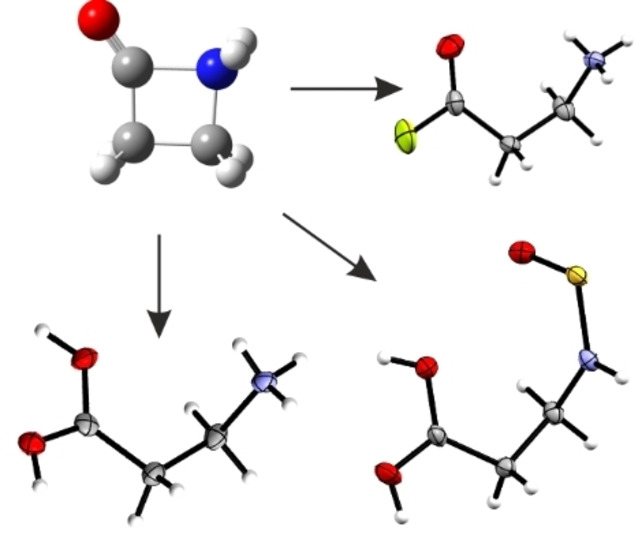

## Introduction

**Scheme 1:**
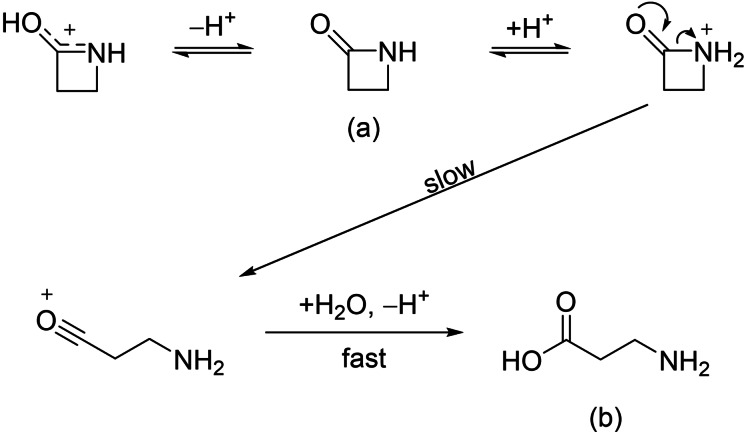
Postulated reaction mechanism of acid‐catalyzed ring opening of β‐propiolactam by Yates.^[4]^

The β‐lactam motif is of particular importance for the eponymous class of antibiotics,^[1]^ of which the best‐known representatives are penicillins and cephalosporins.[^1^, ^2^] The opening of the β‐lactam ring plays a decisive role in the mode of action of these antibiotics and potential resistance to them.^[1]^ Given this importance, many studies on the basic structure of β‐propiolactam have been conducted.[^3^, ^4^, ^5^] In particular, the mechanism and corresponding kinetics of acid‐catalyzed hydrolysis, including ring opening, have been investigated.[^3^, ^4^] For these kinetic measurements, β‐propiolactam (Scheme 1a) was reacted in aqueous sulfuric acid so that the exclusive product of hydrolysis was β‐alanine (Scheme 1 b).[^3^, ^4^] The ring opening is proposed to be subject to a unimolecular mechanism with the formation of an acylium ion as rate‐determining step.^[4]^

For this mechanism the requirement of an N‐protonated species was assumed, but the O‐protonated species was determined to be the major formed intermediate.^[3]^

Focusing on its two basic centers and regarding its hydrolysis behavior, calculations of the gas‐phase basicity of β‐propiolactam were performed.^[5]^ The calculations showed that β‐propiolactam is an oxygen base, with the gap between the intrinsic basicities of oxygen and nitrogen being very small.^[5]^

Taking advantage of the reactivity of β‐propiolactam, Tepe et al. performed trifluoromethanesulfonic acid‐catalyzed Friedel–Crafts acylations.^[6]^ Interestingly, even in superacidic media the same mechanism for the four‐membered ring opening is assumed^[6]^ as described in Scheme 1. This prompted us to study the reaction behavior of β‐propiolactam in the superacidic systems HF/*M*F_5_ (*M*=Sb, As).

## Results and Discussion

### Preparation

β‐Propiolactam reacts in the superacidic system HF/*M*F_5_ (*M*=Sb, As) according to Equations (1) and 2.

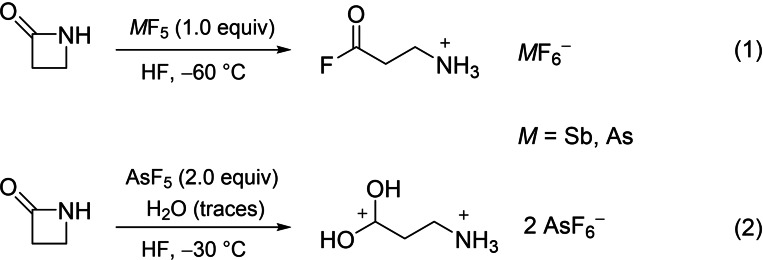




Hydrogen fluoride was used in excess and served as reagent as well as solvent. One or two equivalents of the respective Lewis acid were added and completely solvated by homogenizing the mixture at −40 °C. Under nitrogen atmosphere, β‐propiolactam was added to the frozen system. Using one equivalent Lewis acid and homogenizing the reaction mixture at −60 °C led to the formation of salts of monoprotonated 3‐aminopropanoyl fluoride [Eq. (1)]. As already postulated by Yates et al.,^[4]^ the protonation of β‐propiolactam leads to a ring opening reaction, even in superacidic media. In this context, an acyl cation in the form of [NH_2_(CH_2_)_2_CO][*M*F_6_] is expected to be formed. The formal addition of a HF molecule to the acyl cationic species led to the generation of the air‐ and temperature sensitive compounds [C(O)F(CH_2_)_2_NH_3_][SbF_6_] (**1**) and [C(O)F(CH_2_)_2_NH_3_][AsF_6_] (**2**), which decompose above −10 °C.

Using two equivalents of the Lewis acid arsenic pentafluoride and increasing the reaction temperature to −30 °C, the diprotonated species of β‐alanine, [C(OH)_2_(CH_2_)_2_NH_3_][AsF_6_]_2_ (**3**), was obtained [Eq. (2)]. Based on the assumed acyl cation [NH_2_(CH_2_)_2_CO][*M*F_6_], a formal addition of a water molecule, followed by protonation of the amino group yielded compound **3**. For the formation of this compound small amounts of water are needed. They could be traced back to the use of a non‐anhydrous reaction set up. The temperature‐ and air‐sensitive salt **3** is stable up to 0 °C.

By reacting β‐propiolactam in SO_2_ with two equivalents of HF, and SbF_5_ respectively, the formation of the air‐sensitive salt [C(OH)_2_(CH_2_)_2_NHSO][SbF_6_]_2_⋅HF (**4**) was observed [Eq. 3].

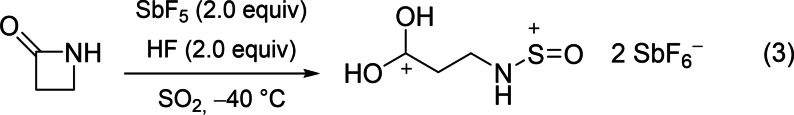




**Scheme 2:**
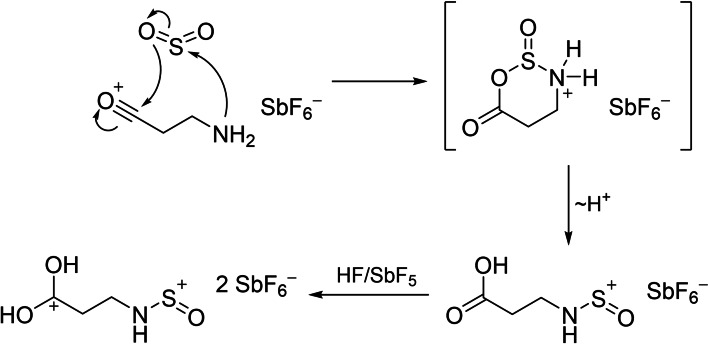
Possible reaction pathway for the formation of **4**.

As the formation process of **4** is not obvious, a possible reaction pathway is given in Scheme 2. In particular, the assumed nucleophilic attack on sulfur suggests the existence of the acyl cationic species [NH_2_(CH_2_)_2_CO][*M*F_6_].

### Crystal structures

#### [C(O)F(CH_2_)_2_NH_3_][SbF_6_] (1)

**Figure 1:**
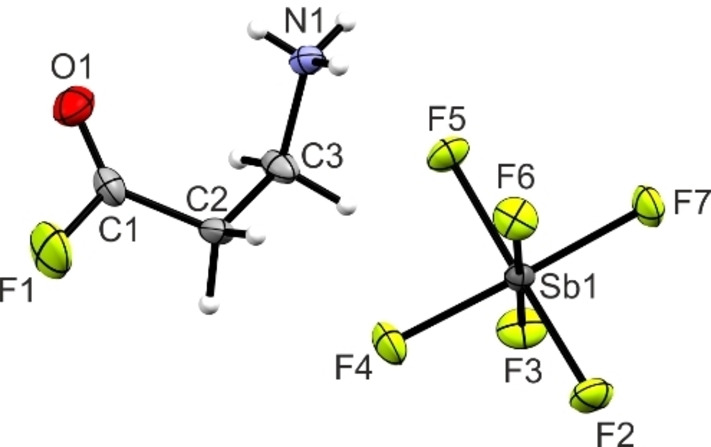
Asymmetric unit of **1** with 50 % probability displacement ellipsoids.

The salt **1** crystallizes in the triclinic space group *P* $\bar{1}$ with two formula units per unit cell. The asymmetric unit of **1** is displayed in Figure 1 and selected bond lengths and angles are summarized in Table 1. Interatomic contacts are illustrated in Figure S1 and the respective values are given in Table S1 (see the Supporting Information).

**Table 1:** Comparison of selected bond lengths and angles of **1**, **3** and **4** with estimated standard deviations in parentheses.

	**1**	**3**	**4**
Bond lengths [Å]			
C1−F1	1.340(6)		
C1−O1	1.176(6)	1.270(3)	1.268(6)
C1−O2		1.264(3)	1.270(6)
C1−C2	1.480(6)	1.481(3)	1.489(8)
C2−C3	1.508(6)	1.518(3)	1.513(6)
C3−N1	1.484(4)	1.498(4)	1.488(7)
N1−S1			1.563(4)
S1−O3			1.431(4)

Bond angles [°]			
O1−C1−F1	119.4(4)		
F1−C1−C2	111.9(3)		
O1−C1−O2		118.4(2)	117.7(5)
O1−C1−C2	128.8(4)	117.7(2)	119.4(5)
O2−C1−C2		123.9(2)	122.9(5)
C1−C2−C3	112.8(3)	114.9(2)	114.2(4)
C2−C3−N1	112.6(3)	112.3(2)	112.4(4)
C3−N1−S1			127.8(4)
N1−S1−O3			111.4(2)

Dihedral angles [°]			
F1−C1−C2−C3	173.9(3)		
O1−C1−C2−C3	−6.4(6)	4.9(3)	−5.6(7)
O2−C1−C2−C3		−174.7(2)	175.2(5)
C1−C2−C3−N1	66.2(4)	76.7(3)	71.0(6)
C2−C3−N1−S1			−115.7(5)
C3−N1−S1−O3			3.1(5)

The C1−O1 bond length measures 1.176(6) Å and is in the typical range for CO bonds in acyl fluoride groups.[^7^, ^8^, ^9^] Moreover, the C1−F1 bond distance of 1.340(6) Å exhibits a characteristic CF bond length in acyl fluoride groups.[^7^, ^8^, ^9^] The C−C bond lengths, with values of 1.480(6) Å (C1−C2) and 1.508(6) Å (C2−C3), are slightly shorter than a formal C−C single bond (1.54 Å).^[10]^ The C3−N1 bond length (1.484(4) Å) is, due to the protonation, slightly longer than a formal C−N bond (1.47 Å)^[10]^ and in accordance with reported C−NH_3_^+^ bond lengths.[^11^, ^12^] The bond angles around the acyl fluoride moiety are in the range between 111.9(3)° (F1−C1−C2) and 128.8(4)° (O1−C1−C2). The molecule skeleton spans an dihedral angle of 66.2(4)° (C1−C2−C3−N1).

The Sb−F bond lengths of the SbF_6_^−^ anion are in the range between 1.865(2) Å (Sb1−F2) and 1.883(2) Å (Sb1−F5). Bond angles are between 87.08(9)° (F5−Sb1−F6) and 91.8(1)° (F3−Sb1−F2) or between 177.4(1)° (F6−Sb1−F2) and 178.4(1)° (F7−Sb1−F4), respectively. All of these values are in accordance with reported literature for SbF_6_^−^ anions.[^13^, ^14^]

#### [C(OH)_2_(CH_2_)_2_NH_3_][AsF_6_]_2_ (3)

**Figure 2:**
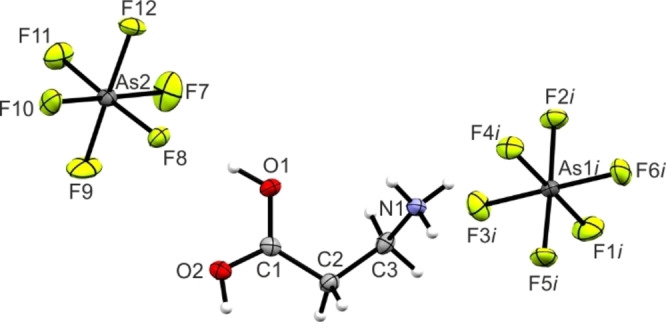
Formula unit of **3** with 50 % probability displacement ellipsoids. Symmetry code: *i*=1/2 −*x*,−1/2 +*y*,1/2 −*z*.

Compound **3** crystallizes in the monoclinic space group *P*2_1_/*n* with four formula units per unit cell. In Figure 2 the formula unit is illustrated and in Table 1 selected bond lengths and angles are listed. Interatomic contacts are displayed in Figure S2 and corresponding values are listed in Table S2.

In **3** both C−O bond lengths (C1−O1: 1.270(3) Å, C1−O2: 1.264(3) Å) are between a formal single (1.43 Å)^[10]^ and double bond (1.19 Å).^[10]^ The angles around the protonated acid moiety are between 117.7(2)° (O1−C1−C2) and 123.9(2)° (O2−C1−C2). These values are in good agreement with those reported in literature for this moiety.[^15^, ^16^] The C1−C2 bond (1.481(3) Å) next to the protonated acid group is comparable with the C1−C2 bond length in **1** (1.480(6) Å). In contrast, the bond lengths of C2−C3 (1.518(3) Å) and C3−N1 (1.498(4) Å) are slightly elongated compared to **1**. With a value of 76.7(3)°, the dihedral angle C1−C2−C3−N1 of **3** is about 10° wider than in **1**.

The As−F bond lengths of the two AsF_6_^−^ anions are between 1.693(2) Å (As2−F9) and 1.748(2) Å (As2−F8) and in the typical range for AsF_6_^−^ anions.[^13^, ^14^, ^17^] Additionally, the F−As−F angles, which are in the range between 87.9(8)° (F8−As2−F12) and 92.5(9)° (F11−As2−F10), and between 175.6(1)° (F10−As2−F7) and 179.0(9)° (F3−As1−F6), are in accordance with reported AsF_6_^−^ anions.[^13^, ^14^, ^17^]

#### [C(OH)_2_(CH_2_)_2_NHSO][SbF_6_]_2_⋅HF (4)

**Figure 3:**
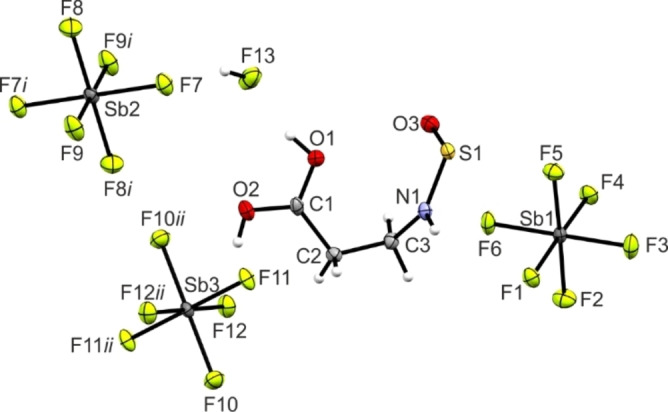
Formula unit of **4** with 50 % probability displacement ellipsoids. Symmetry codes: *i*=2−*x*,−*y*,2−*z*; *ii*=2−*x*,−*y*,1−*z*.

Salt **4** crystallizes in the triclinic space group *P* $\bar{1}$ with two units per unit cell. In Figure 3, the formula unit of **4** is displayed and in Table 1 selected bond lengths and angles of **4** together with those of **1** and **3** are summarized. A projection of interatomic contacts of **4** is illustrated in Figure S3 and respective values are listed in Table S3.

Both C−O bond lengths of **4** (C1−O1: 1.268(6) Å, C1−O2: 1.270(6) Å) are closely comparable with those of **3**, as the C1−O1 and C1−O2 bond lengths are not significantly different. The same accordance is observed for both C−C bonds (1.489(8) Å (C1−C2), 1.513(6) Å (C2−C3)) as well as for the C3−N1 bond, with a value of 1.488(7) Å. The bond angles of **4** are in good agreement with those of **3**, as their molecular skeletons are comparable (Table 1).

The bond lengths of the thionylimide moiety (HNSO) are comparable with reported crystal data for H−NSO trapped by adduct formation with bulky Lewis acids.^[18]^ The N1−S1 bond length of **4**, with a value of 1.563(4) Å, is significantly longer than the bond length of the free thionylimide (1.530(2) Å).^[18]^ Both bond lengths are between a formal S−N single (1.73 Å) and double bond (1.49 Å).^[10]^ In contrast, greater agreement between **4** and the free thionylimide is observed for the S1−O3 bond (1.431(4) Å (**4**) and 1.427(2) Å^[18]^). These bond lengths are in the range of SO double bonds (S=O: 1.46 Å, S=2O: 1.34 Å).^[10]^ The N1−S1−O3 angle 111.4(2)° is comparable to that observed in the free HNSO (114.3(2)°).^[18]^

The Sb−F bond lengths are between 1.860(3) Å (Sb2−F8) and 1.891(3) Å (Sb2−F7). F−Sb−F angles are between 88.5(1)° (F4−Sb1−F2) and 92.8(1)° (F5−Sb1−F4) and between 178.1(1)° (F4−Sb1−F1) and 180.0(1)° (F11−Sb3−F11*ii*), respectively. All bond lengths and angles found in both SbF_6_^−^ anions are in good agreement with data reported in literature.[^13^, ^14^]

### Vibrational spectroscopy

#### Monoprotonated 3‐aminopropanoyl fluoride

**Figure 4:**
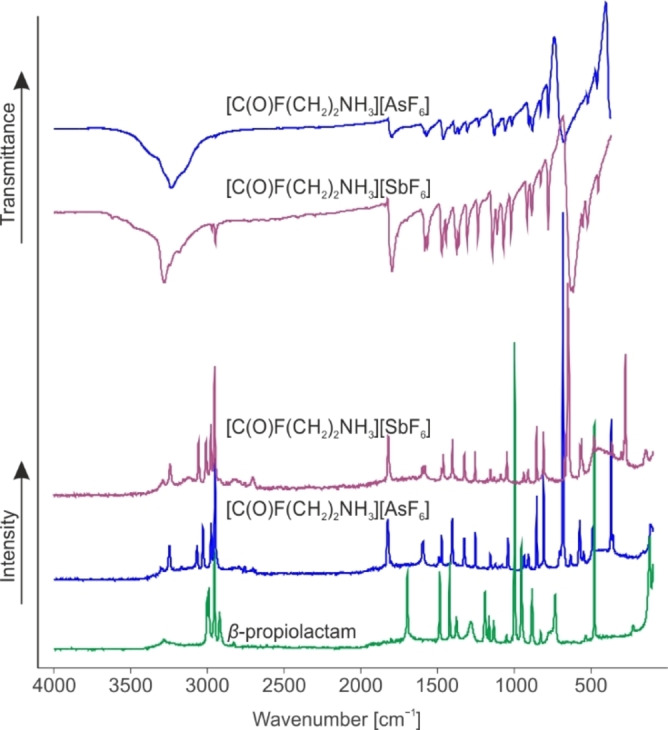
Raman spectrum of β‐propiolactam (green), Raman and IR spectra of **1** (violet) and **2** (blue).

The infrared and Raman spectra of **1** and **2** together with the Raman spectrum of β‐propiolactam are displayed in Figure 4.

Selected experimental and calculated vibrational frequencies are listed in Table 2. The [C(O)F(CH_2_)_2_NH_3_]^+^ cation possesses *C*_1_ symmetry with 33 fundamental vibrations, active in both Raman and infrared spectra. A complete list of all experimentally obtained and calculated frequencies is given in Table S4. For better accordance of the calculated and experimental frequencies one HF molecule was added to the gas phase structure, to simulate the hydrogen bonding in the solid state. The most characteristic vibration of β‐propiolactam, the ring breathing vibration at 962 cm^−1^,^[19]^ is not detectable in the Raman spectra of **1** and **2**. Moreover, the CO stretching vibration is blue‐shifted compared to the starting material^[19]^ and occurs in the range between 1812 cm^−1^ (IR of **1**) and 1826 cm^−1^ (Ra of **2**). This range for *ν*(CO) is typical for acyl fluoride groups.[^20^, ^21^] At 1157 cm^−1^ in both Raman spectra and at 1165 (**1**) and 1155 cm^−1^ (**2**) in the IR spectra, the corresponding *ν*(CF) vibrations are observed. For the NH_3_ group three NH stretching vibrations are expected. They occur in the range between 3158 (IR of **2**) and 3303 cm^−1^ (Ra of **2**). The *ν*(CN) vibration of the formed protonated primary amine is detected at about 856 cm^−1^ for **1** and **2**. However, the typical range for primary amines is between 1030 and 1090 cm^−1^.^[22]^ This red‐shift can be explained by the protonation and is in accordance with reported literature data of protonated primary amines.^[11]^ To conclude, the vibrational spectroscopy confirms the results of the X‐ray diffraction analysis.

**Table 2:** Selected experimental vibrational frequencies [cm^−1^] of **1** and **2** and calculated vibrational frequencies [cm^−1^] of [C(O)F(CH_2_)_2_NH_3_]^+^ **⋅**HF.

**1**		**2**		[C(O)F(CH_2_)_2_NH_3_]^+^ **⋅**HF	Assignment
IR	Raman	IR	Raman	Calc.^[a]^ (IR/Raman)	
3283 (w)	3290 (8)	3303 (w, sh)	3302 (3)	3482 (122/56)	*ν*_as_(NH_3_)
3244 (w)	3244 (15)	3238 (m)	3247 (9)	3355 (381/71)	*ν*_as_(NH_3_)
3186 (vw)		3158 (w, sh)	3166 (4)	3242 (203/54)	*ν*_s_(NH_3_)
1812 (w)	1822 (28)	1814 (vw)	1826 (17)	1842 (228/13)	*ν*(CO)
1165 (w)	1157 (13)	1155 (w)	1157 (7)	1208 (174/2)	*ν*(CF)
1045 (w)	1050 (21)	1041 (vw)	1043 (12)	1024 (9/2)	*ν*(CC)
912 (vw)	913 (13)	908 (w)	910 (7)	917 (37/1)	*ν*(CC)
854 (vw)	857 (33)	854 (w)	856 (23)	843 (2/8)	*ν*(CN)

[a] Calculated at the B3LYP/aug‐cc‐pVTZ level of theory. IR intensity in km/mol and Raman intensity in Å^4^/u. Abbreviations for IR intensities: v=very, m=medium, w=weak. Experimental Raman activities are stated to a scale of 1 to 100.

For both anions, SbF_6_^−^ and AsF_6_^−^, more vibrations than expected for ideal *O*_h_ symmetry are observed. In the Raman spectra of **1** and **2** more than three lines and in the corresponding infrared spectra more than two bands are detected. The increased number of vibrations indicates a lowered symmetry of the anion structures, which is in accordance with the results of the X‐ray study.

#### Diprotonated β‐alanine

**Figure 5:**
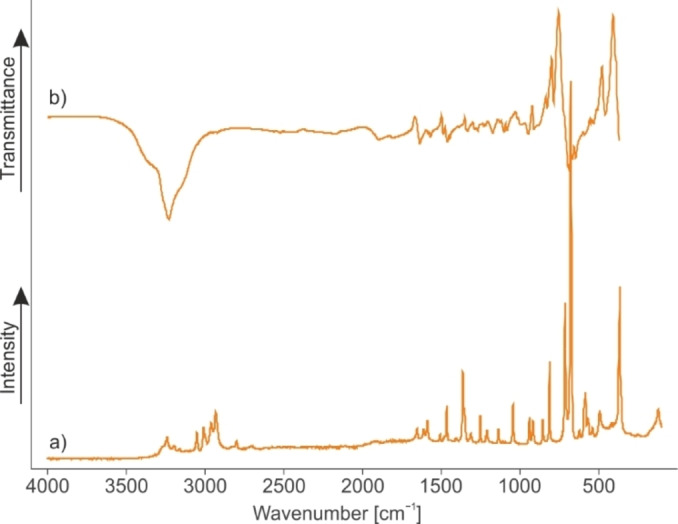
a) Raman and b) infrared spectra of **3**.

In Figure 5, the Raman and infrared spectra of diprotonated β‐alanine in the form of **3** are illustrated. Table 3 summarizes selected calculated and observed vibrational frequencies of **3** together with experimental frequencies of the neutral compound β‐alanine for comparison.^[23]^ The complete table is given in the Supporting Information (Table S5). In order to simulate the interatomic contacts in the solid state, three HF molecules were added to the calculated gas phase structure.

**Table 3:** Selected experimental vibrational frequencies [cm^−1^] of **3** and calculated vibrational frequencies [cm^−1^] of [C(OH)_2_(CH_2_)_2_NH_3_]^2+^ **⋅**3 HF.

**3**		[C(OH)_2_(CH_2_)_2_NH_3_]^2+^ **⋅**3 HF	β‐Alanine^[23]^	Assignment
IR	Raman	Calcd.^[a]^ (IR/Raman)		
3234 (vs)	3242 (6)	3467 (103/17)	3408	*ν*_as_(NH_3_)/*ν*_as_(NH_2_)^[23]^
3159 (vs, sh)	3161 (3)	3206 (544/156)		*ν*_s_(NH_3_)/*ν*_as_(NH_2_)^[23]^
3125 (s,sh)		3161 (1589/121)	3559	*ν*(OH)
1605 (w)		1653 (310/0.7)	1770	*ν*(CO)
1589 (w)	1590 (11)	1568 (212/5)	1159	*ν*(CO)
1312 (w)	1316 (8)	1329 (111/0.2)	1267	*δ*(COH)
1196 (w)	1214 (8)	1275 (214/5)		*δ*(COH)
856 (m)	858 (10)	841 (11/3)	1050	*ν*(CN)

[a] Calculated at the B3LYP/aug‐cc‐pVTZ level of theory. IR intensity in km/mol and Raman intensity in Å^4^/u. Abbreviations for IR intensities: v=very, s=strong, m=medium, w=weak, sh=shoulder. Experimental Raman activities are stated to a scale of 1 to 100.

For the cation, *C*_1_ symmetry with 36 fundamental vibrations, active in Raman and IR, is expected. The first evidence for the diprotonated β‐alanine is the *ν*(CO) vibration at 1605 cm^−1^ (IR). This vibration is red‐shifted by approximately 165 cm^−1^ compared to the neutral compound.^[23]^ As β‐alanine turned out to be a nitrogen base and the N‐protonated species is already known,^[12]^ this shift indicates a second protonation on the oxygen. The *ν*(CN) vibration, which occurs at 856 (IR) and 858 cm^−1^ (Ra), shows the same trend, with a red‐shift of about 194 (IR) and 192 cm^−1^ (Ra). Furthermore, the *ν*(OH) vibrations are detected as broad bands at about 3125 cm^−1^ (IR). Due to the poor polarizability of this bond, no lines are observed in the corresponding Raman spectra. In contrast, the NH stretching vibrations occur in the IR and Raman spectra in the range between 3159 and 3242 cm^−1^. The second CO stretching vibration occurs at 1589 (IR) and 1590 cm^−1^ (Ra). These values are in good agreement with reported literature data for protonated carbon acid groups.[^15^, ^16^]

For the AsF_6_^−^ anion, more than three lines in the Raman spectrum and more than two bands in the IR spectrum are observed, which would be the numbers of vibrations for an ideal *O*_h_ symmetry. The increased number of vibrations indicates a lowered symmetry for the structure of this anion and is in accordance with X‐ray diffraction analysis.

### Theoretical calculations

**Scheme 3:**
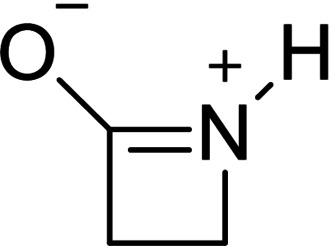
Lewis structure of amide resonance structure of β‐propiolactam.

In this study, the performed experiments led exclusively to the formation of open‐chained compounds. Herein, it is assumed that the protonated species of β‐propiolactam is necessary for the ring opening reaction. Gas‐phase calculations on whether β‐propiolactam is an oxygen or nitrogen base were carried out with the result that the protonation will preferably occur on the oxygen atom.^[5]^ However, the gap between oxygen and nitrogen intrinsic basicities is very small.^[5]^ This can be explained by the lack of amide resonance (Scheme 3), due to the steric strain in the four‐membered ring.

**Figure 6:**
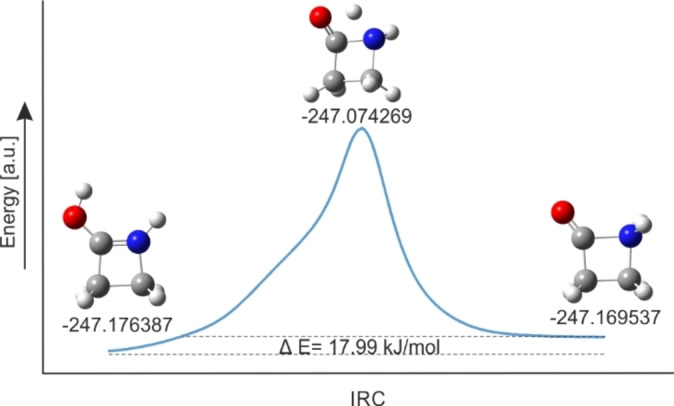
Calculated intrinsic reaction coordinate (IRC) path of the proton shift from O‐protonated to N‐protonated β‐propiolactam together with respective single‐point energy values.

To elucidate if a proton transfer (from oxygen to nitrogen) is likely, the intrinsic reaction coordinate (IRC) path was calculated for β‐propiolactam at the MP2/aug‐cc‐pVTZ level of theory.^[24]^ In Figure 6, the optimized structures of the O‐ and N‐protonated species, the transition state and the calculated intrinsic reaction coordinate path of the possible proton shift is displayed. The 1,3‐proton shift for β‐propiolactam is calculated to be endothermic (+17.99 kJ mol^−1^) in the gas phase. This result leads to the assumption that a direct N‐protonation appears more likely than the proton transfer. As there is no clear contradiction to the calculated intrinsic basicities,^[5]^ natural bond orbital (NBO) analyses were performed to gain closer insights into the different species.^[24]^ In Table S6–S8 selected NBOs together with calculated values for occupancy and s‐ and p‐character of the different species are summarized. Additionally, a comparison of the most meaningful NBOs is given in Table S9.

**Figure 7:**
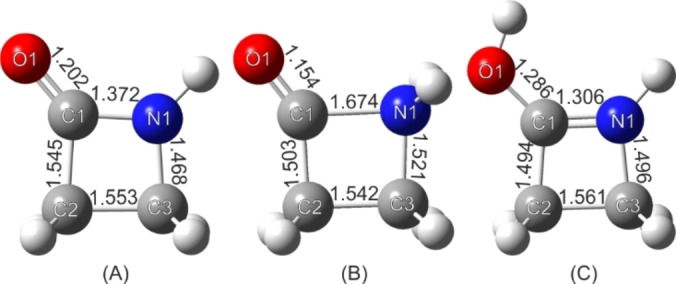
Optimized structures of the A) β‐propiolactam, B) N‐protonated species and C) O‐protonated species, together with calculated bond lengths at the B3LYP/aug‐cc‐pVTZ level of theory.

The optimized structures of β‐propiolactam, and both the O‐ and N‐protonated species are illustrated in Figure 7 together with calculated bond lengths.

**Figure 8:**
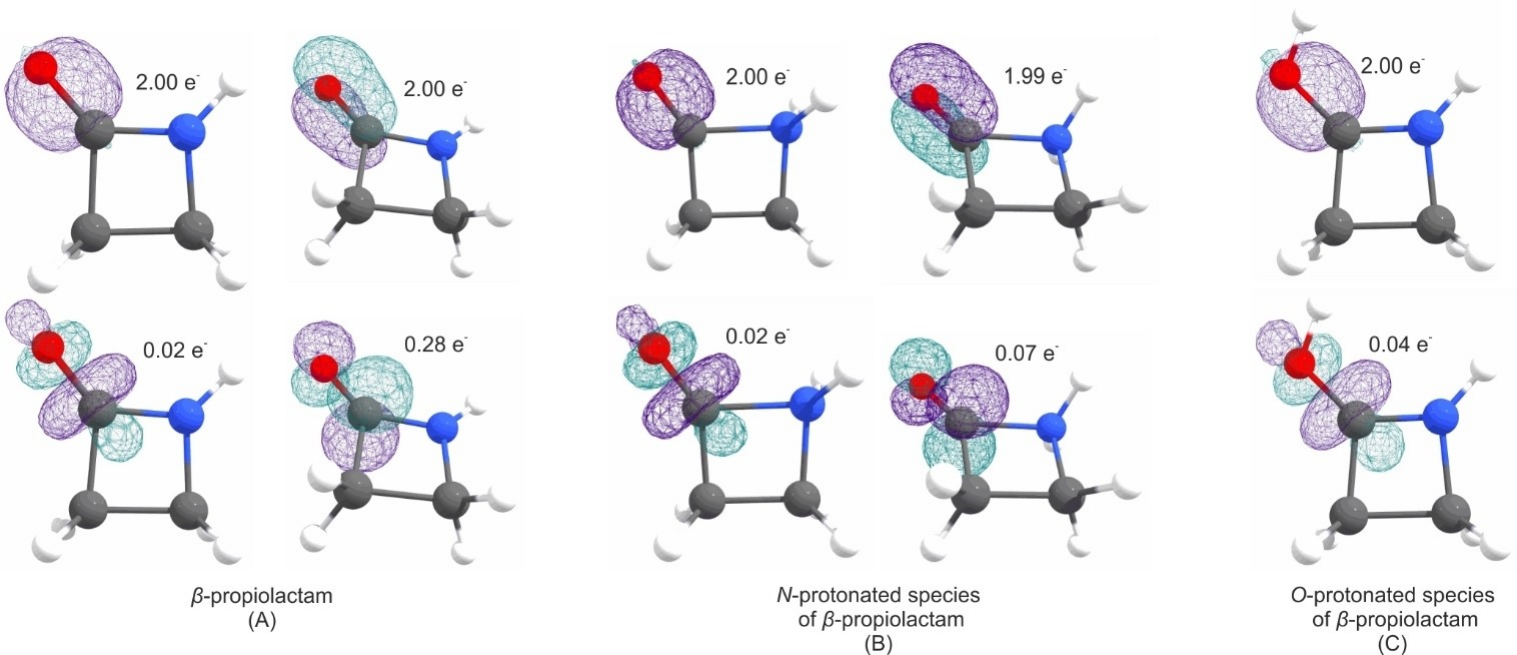
Selected NBOs for the CO bond with corresponding occupancies of the A) β‐propiolactam, B) N‐protonated species and C) O‐protonated species.

Due to the respective protonation, both the C1−O1 and C1−N1 bonds are affected the most. In Figure 8, the calculated NBOs for the C−O bond of the different species are displayed. Concerning the C1−O1 bond in β‐propiolactam (A), two types of bonds (one σ and one π bond) are calculated, both occupied with 2.00 e^−^. The corresponding antibonding orbitals are occupied with 0.02 e^−^ (σ* bond) and 0.28 e^−^ (π* bond). Compared to that, in the N‐protonated species (B) (also one σ and one π bond), the s character of the σ bond increases while the p character decreases. Moreover, the according π* bond is less occupied than in the neutral compound, with only 0.07 e^−^. These two results lead to the conclusion that the bond strength of the C1−O1 bond increases in the N‐protonated species, which is confirmed by the calculated shortened bond length (1.154 Å). In the O‐protonated species only one σ bond with less s‐character than in the two other structures is calculated. This bond is weakened due to the protonation, as it is expected.

**Figure 9:**
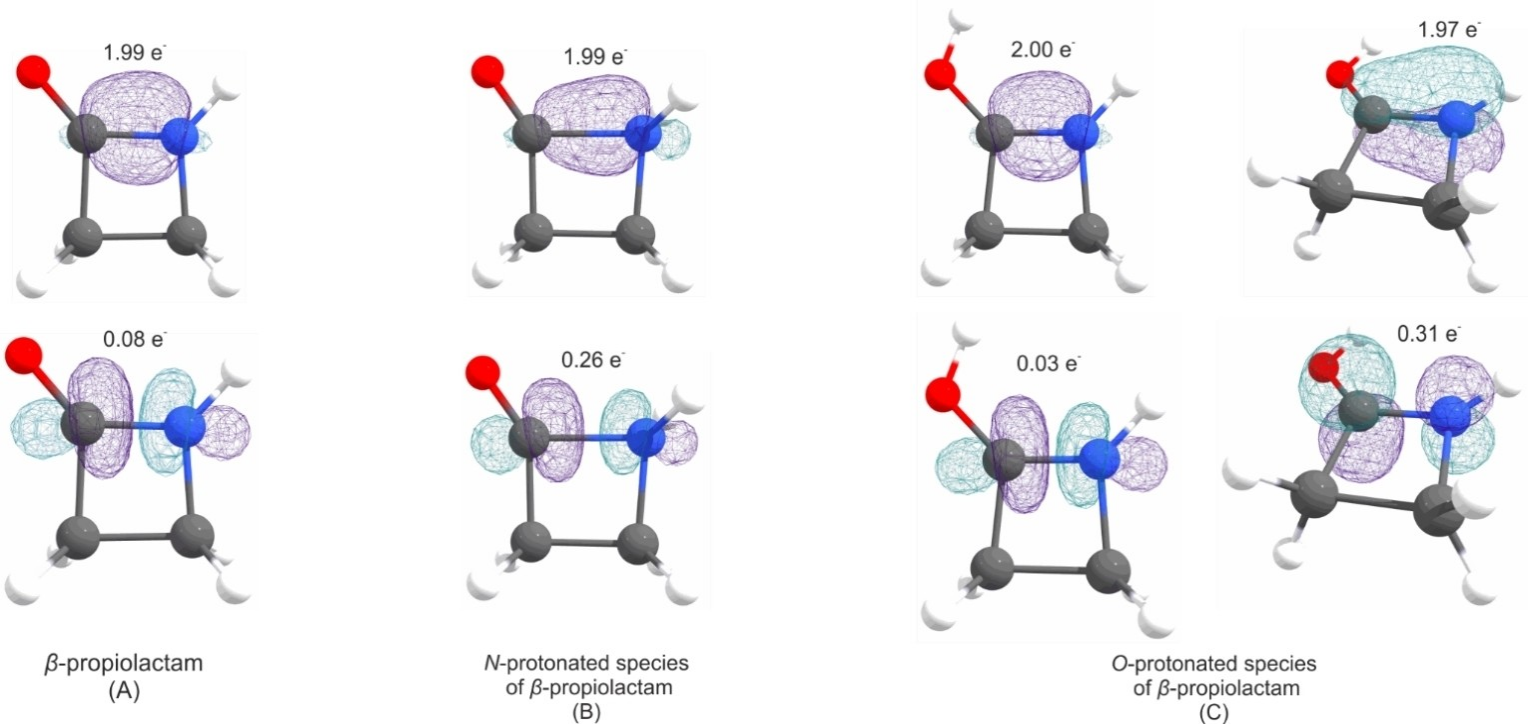
Selected NBOs for the CN bond with the corresponding occupancies of the A) β‐propiolactam, B) N‐protonated species and C) O‐protonated species.

The situation is different for the C1−N1 bond. Selected NBOs for the CN bond are illustrated in Figure 9. In the neutral compound (A) the calculation revealed only one σ bond for the C1−N1 bond, whereas two bonds (one σ and one π bond) are determined by the calculation for the O‐protonated species (C). The s character of the σ bond herein is slightly increased, compared to β‐propiolactam. Moreover, corresponding antibonding orbitals are calculated to be occupied with 0.03 e^−^ (σ* bond) and 0.31 e^−^ (π* bond). The calculation results show that this bond is strengthened, which is supported by the bond length of 1.306 Å. In summary, O‐protonation leads to a strengthening of the C1−N1 bond and makes ring opening impossible. In the N‐protonated species (B), the calculation revealed only one σ bond, where the s character is about 20 %. Compared to the other two structures, the s character is decreased while the p character is increased. Additionally, the calculation shows that the corresponding σ* orbital is, with 0.26 e^−^, more strongly occupied than the O‐protonated species (C). In conclusion, these values show that the strength of the C1−N1 bond is not only decreased in this species, but it also turned out to be a very weak σ bond in general. As these calculations clarify the bonding situation in protonated β‐propiolactam, the N‐protonated species turned out to be the only species, which is able to form the acyl cation [NH_2_(CH_2_)_2_CO]^+^.

## Conclusion

In this study, the reaction behavior of β‐propiolactam in the superacidic systems HF/*M*F_5_ (*M*=Sb, As) was investigated. With an equimolar amount of Lewis acid, the salts of monoprotonated 3‐aminopropanoyl fluoride [C(O)F(CH_2_)_2_NH_3_][SbF_6_] (**1**) and [C(O)F(CH_2_)_2_NH_3_][AsF_6_] (**2**) were obtained. Changing the reaction conditions to a larger amount of Lewis acid together with traces of water, led to the formation of the hitherto unknown diprotonated species of β‐alanine [C(OH)_2_(CH_2_)_2_NH_3_][AsF_6_]_2_ (**3**). The salts were characterized by Raman and IR spectroscopy, and, in the case of **1** and **3**, single‐crystal X‐ray analyses were performed. By changing the solvent from HF to SO_2_, the salt [C(OH)_2_(CH_2_)_2_NHSO][SbF_6_]_2_ **⋅**HF (**4**), which includes a thionylimide moiety, was formed, as detected by single‐crystal X‐ray analysis. Experimental evidence of these open‐chain compounds suggests that they might be formed via the same reactive intermediate [NH_2_(CH_2_)_2_CO][*M*F_6_]. This species is proposed to form from N‐protonated β‐propiolactam. As a protonated cyclic species (O‐ or N‐protonated) of the oxygen base β‐propiolactam was neither isolated nor observed. Theoretical calculations were also performed. The calculated intrinsic reaction coordinate path shows that an intramolecular proton shift from oxygen to nitrogen is unlikely, because of its endothermic character (+17.99 kJ mol^−1^) in the gas phase. Subsequently, natural bond orbital analyses were performed to get closer insights into the different protonated species. The analyses could support the assumption that the N‐protonated species is responsible for the formation of the highly reactive acyl cationic species.

## Experimental Section

### General

***CAUTION***! Avoid contact with any of these compounds. Note that hydrolysis of AsF_5_, SbF_5_ and the prepared salts might form HF, which burns skin and causes irreparable damage. Safety precautions should be taken while using and handling these materials.

**Apparatus and materials**: All reactions were conducted by employing standard Schlenk techniques using a stainless‐steel vacuum line. FEP/PFA reactors, closed with a stainless steel valve, were used to perform all reactions in superacidic media. Prior to use, all reaction vessels and the stainless steel vacuum line were dried with fluorine (excluding reactions to obtain compound (**3**)). IR spectroscopic investigations were performed on a Vertex‐80 V FTIR spectrometer (*ṽ*=350–4000 cm^−1^) by placing small amounts of the respective sample on a CsBr single‐crystal plate in a cooled cell. Raman measurements were carried out on a Bruker MultiRAM FT‐Raman spectrometer with Nd:YAG laser excitation (*λ*=1064 cm^−1^) under vacuum at −196 °C. For measurements, the synthesized compounds were transferred into a cooled glass cell. The low‐temperature single‐crystal X‐ray diffraction of **1**, **3** and **4** were performed on an Oxford XCalibur 3 diffractometer equipped with a Kappa CCD detector operating with Mo_Κα_ radiation (*λ*=0.71073 Å) and a Spellman generator (voltage 50 kV, current 40 mA). The program CrysAlisPro 1.171.38.46 (Rigaku OD, 2015)^[25]^ was employed for the data collection and reduction. The structures were solved utilizing SHELXT^[26]^ and SHELXL‐2018/3^[27]^ of the WINGX software package.^[28]^ The structures were checked using the software PLATON.^[29]^ The absorption correction was performed using the SCALE3 ABPSACK multiscan method.^[30]^ selected crystallographic parameters and data for **1**, **3** and **4** are listed in Table S10. The quantum chemical calculations were performed at the B3LYP/aug‐cc‐pVTZ and MP2/aug‐cc‐pVTZ levels of theory.

**Synthesis [C(O)F(CH_2_)_2_NH_3_][SbF_6_] (1) and [C(OH)_2_(CH_2_)_2_NHSO][SbF_6_]_2_ (4)**: Antimony pentafluoride (160 mg, 0.74 mmol, 1.0 equiv. for **1** and 310 mg, 1.43 mmol, 2.0 equiv. for **4**) was condensed at −196 °C into a FEP tube reactor. Afterwards, anhydrous hydrogen fluoride (*a*HF, approximately 2 mL for **1** and 28.6 mg, 1.43 mmol, 2.0 equiv. for **4**) was condensed into the reactor under the same conditions. In the case of **4**, sulfur dioxide (2 mL) was condensed additionally at −196 °C. In order to form the superacidic system, the respective compounds were warmed up to −40 °C and homogenized. The mixture was cooled again to −196 °C and subsequently β‐propiolactam (52 mg, 0.74 mmol, 1.0 equiv. (**1**) and 51 mg, 0.72 mmol, 1.0 equiv. (**4**)) was added under nitrogen atmosphere. The reaction mixture was warmed up to −60 (**1**) or −40 °C (**4**) and homogenized until the respective salts were completely dissolved. For crystallization of compounds **1** and **4**, the reactors were left in an ethanol bath at −70 (**1**), or −50 °C (**4**) until the salts recrystallized. For Raman und IR measurements of **1**, the reactor was cooled down to −196 °C again and excess *a*HF was removed at −78 °C in a dynamic vacuum overnight. Compound **1** was obtained as a colorless solid, which is stable up to −10 °C.

**Synthesis of [C(O)F(CH_2_)_2_NH_3_][AsF_6_] (2)**: Anhydrous hydrogen fluoride, approximately 2 mL, was condensed into a FEP tube reactor at −196 °C. Subsequently, arsenic pentafluoride (85 mg, 0.5 mmol, 1.0 equiv.) was condensed into the reactor under the same conditions. To form the superacidic system, the compounds were warmed up to −40 °C and homogenized. After cooling down to −196 °C again, β‐propiolactam (35 mg, 0.5 mmol, 1.0 equiv.) was added under nitrogen atmosphere. For homogenization, the reaction mixture was warmed up to −60 °C. After cooling down to −196 °C again, excess *a*HF was removed overnight in a dynamic vacuum. Compound **2** was obtained as a colorless solid, which is stable up to −10 °C.

**Synthesis of [C(OH)_2_(CH_2_)_2_NH_3_][AsF_6_]_2_ (3)**: As a modification to the reaction procedure of **2**, compound **3** was obtained by using a non‐anhydrous reaction set up. Anhydrous hydrogen fluoride (2 mL) and arsenic pentafluoride (170 mg, 1.0 mmol, 2.0 equiv.) were condensed into a FEP tube reactor at −196 °C. Both compounds were warmed up to −30 °C and homogenized. After cooling down again to −196 °C β‐propiolactam (35 mg, 0.5 mmol, 1.0 equiv.) was added under nitrogen atmosphere. The reaction mixture was warmed up again to −30 °C and homogenized until the salt was completely dissolved. The mixture was cooled down to −196 °C and excess *a*HF was removed overnight in a dynamic vacuum. Compound **3** was obtained as a colorless solid, with a decomposition temperature of 0 °C. To crystallize compound **3**, the reactor was left in an ethanol bath at −40 °C until the salt recrystallized.

**Crystallographic data**: Deposition Numbers 2062961 (for **1**), 2062962 (for **3**) and 2062963 (for **4**) contain the supplementary crystallographic data for this paper. These data are provided free of charge by the joint Cambridge Crystallographic Data Centre and Fachinformationszentrum Karlsruhe Access Structures service.

## Conflict of interest

The authors declare no conflict of interest.

1

## Supporting information

As a service to our authors and readers, this journal provides supporting information supplied by the authors. Such materials are peer reviewed and may be re‐organized for online delivery, but are not copy‐edited or typeset. Technical support issues arising from supporting information (other than missing files) should be addressed to the authors.

Supporting Information

## Data Availability

The data that support the findings of this study are available in the supplementary material of this article.
