# Supplementary material for: Ring Opening Reactions of β‐Propiolactam in Superacidic Media
Source: Chemistry. 2022 Jan 5;28(6):e202104086. doi: 10.1002/chem.202104086 (PMC9302645; doi:10.1002/chem.202104086)
Supplement: Supplementary file 1 — Supporting Information [file CHEM-28-0-s001.pdf]

Chemistry–A European Journal

Supporting Information

Ring Opening Reactions of β-Propiolactam in Superacidic Media

Stefanie Beck, Vanessa Rück, Lea-Viktoria Pietsch, Christoph Jessen, and Andreas J. Kornath*

Table of Contents

Figure S1. Projection of interatomic contacts in the $[C(O)F(CH_2)_2NH_3][SbF_6]$ (**1**) crystal (50% probability displacement ellipsoids).Symmetry code: *i* = x, −1+y, z.

Table S1. Selected bond lengths [Å] and angles [°] of $[C(O)F(CH_2)_2NH_3][SbF_6]$ (**1**) with estimated standard deviations in parentheses. Symmetry code: *i* = x, −1+y, z.

Figure S2. Projection of interatomic contacts in the $[C(OH)_2(CH_2)_2NH_3][AsF_6]_2$ (**3**) crystal (50% probability displacement ellipsoids). Symmetry codes: *i* = ½-x, −½+y, ½−z; *ii* = −1+x, y, z.

Table S2. Selected bond lengths [Å] and angles [°] of $[C(OH)_2(CH_2)_2NH_3][AsF_6]_2$ (**3**) with estimated standard deviations in parentheses. Symmetry codes: *i* = ½−x, −½+y, ½−z; *ii* = −1+x, y, z.

Figure S3. Projection of interatomic contacts in the $[C(OH)_2(CH_2)_2NHSO][SbF_6]_2 \cdot HF$ (**4**) crystal (50% probability displacement ellipsoids). Symmetry code: *i* = 1−x,1−y, 2−z.

Table S3. Selected bond lengths [Å] and angles [°] of $[C(OH)_2(CH_2)_2NHSO][SbF_6]_2 \cdot HF$ (**4**) with estimated standard deviations in parentheses. Symmetry codes: *i* = 1−x,1−y, 2−z.

Table S4. Experimental vibrational frequencies [cm^{-1}] of (**1**) and (**2**) and calculated vibrational frequencies [cm^{-1}] of $[C(O)F(CH_2)_2NH_3]^{+} \cdot HF$.

Table S5. Experimental vibrational frequencies [cm^{-1}] of (**3**) and calculated vibrational frequencies [cm^{-1}] of $[C(OH)_2(CH_2)_2NH_3]^{2+} \cdot 3HF$.

Table S6. Selected NBOs (BD = 2-center bond; LP = 1-center valence lone pair; BD* = 2-center antibond) of *β*-propiolactam together with calculated values for occupancy and s- and p-character.

Table S7. Selected NBOs (BD = 2-center bond; LP = 1-center valence lone pair; BD* = 2-center antibond) of the *N*-protonated species of *β*-propiolactam together with calculated values for occupancy and s- and p-character.

Table S8. Selected NBOs (BD = 2-center bond; LP = 1-center valence lone pair; BD* = 2-center antibond) of the *O*-protonated species of *β*-propiolactam together with calculated values for occupancy and s- and p-character.

Table S9. Comparison of selected NBOs together with electron occupancies (occ.) and s- and p-character of *β*-propiolactam, the *N*-protonated species and the *O*-protonated species of *β*-propiolactam.

Table S10. Crystal data and structure refinement for $[C(O)F(CH_2)_2NH_3][SbF_6]$ (**1**), $[C(OH)_2(CH_2)_2NH_3][AsF_6]_2$ (**3**) and $[C(OH)_2(CH_2)_2NHSO][SbF_6]_2 \cdot HF$ (**4**).

Table S11. Cartesian coordinates of calculated minimum structure of $[C(O)F(CH_2)_2NH_3]^{+} \cdot HF$ on the B3LYP/aug-cc-pVTZ level of theory.

Table S12. Cartesian coordinates of calculated minimum structure of $[C(OH)_2(CH_2)_2NH_3]^{2+} \cdot 3HF$ on the B3LYP/aug-cc-pVTZ level of theory.

Table S13. Cartesian coordinates of calculated minimum structure of the transition state and concurrently starting point for IRC calculations on the MP2/aug-cc-pVTZ level of theory.

Table S14. Cartesian coordinates of calculated minimum structure of endpoint of IRC calculation (*O*-protonation) on MP2/aug-cc-pVTZ level of theory.

Table S15. Cartesian coordinates of calculated minimum structure of endpoint of IRC calculation (*N*-protonation) on MP2/aug-cc-pVTZ level of theory.

Table S16. Cartesian coordinates of calculated minimum structure of *β*-propiolactam on the B3LYP/aug-cc-pVTZ level of theory.

Table S17. Cartesian coordinates of calculated minimum structure of the *N*-protonated species of *β*-propiolactam on the B3LYP/aug-cc-pVTZ level of theory.

Table S18. Cartesian coordinates of calculated minimum structure of the *O*-protonated species of *β*-propiolactam on the B3LYP/aug-cc-pVTZ level of theory.

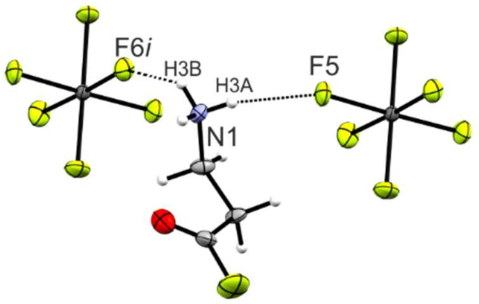

Figure S1. Projection of interatomic contacts in the [C(O)F(CH$_2$)$_2$NH$_3$][SbF$_6$] (**1**) crystal (50% probability displacement ellipsoids).Symmetry code: *i* = x, −1+y, z.

Table S1. Selected bond lengths [Å] and angles [°] of [C(O)F(CH$_2$)$_2$NH$_3$][SbF$_6$] (**1**) with estimated standard deviations in parentheses. Symmetry code: *i* = x, −1+y, z.

Bond lengths [Å]			
N1–C3	1.484(4)	C1–F1	1.340(6)
C3–C2	1.508(6)	C1–O1	1.176(6)
C2–C1	1.480(6)		
Bond angles [°]			
N1–C3–C2	112.6(3)	C2–C1–O1	128.8(4)
C1–C2–C3	112.8(3)	O1–C1–F1	119.4(4)
C2–C1–F1	111.9(3)		
Dihedral angles [°]			
N1–C3–C2–C1	66.2(4)	C3–C2–C1–O1	−6.4(6)
C3–C2–C1–F1	173.9(3)		
Interatomic contacts [Å]			
N1–H3A···F5	2.877(4)	N1–H3B···F6*i*	2.894(5)

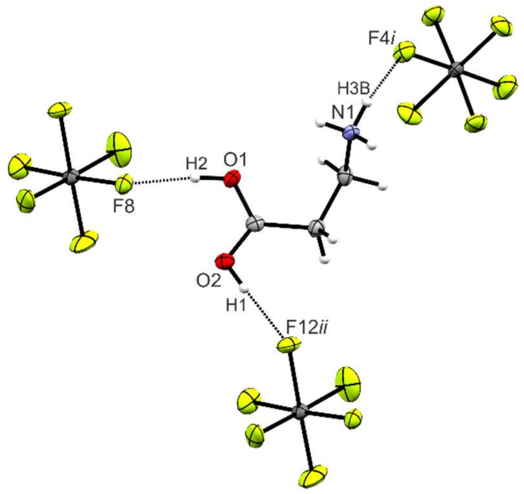

Figure S2. Projection of interatomic contacts in the $[C(OH)_2(CH_2)_2NH_3][AsF_6]_2$ (**3**) crystal (50% probability displacement ellipsoids). Symmetry codes: *i* = ½-x, −½+y, ½−z; *ii* = −1+x, y, z.

Table S2. Selected bond lengths [Å] and angles [°] of $[C(OH)_2(CH_2)_2NH_3][AsF_6]_2$ (**3**) with estimated standard deviations in parentheses. Symmetry codes: *i* = ½−x, −½+y, ½−z; *ii* = −1+x, y, z.

Bond lengths [Å]			
N1–C3	1.498(4)	C1–O1	1.270(3)
C2–C3	1.518(3)	C1–O2	1.264(3)
C1–C2	1.481(3)		
Bond angles [°]			
N1–C3–C2	112.3(2)	O1–C1–O2	118.4(2)
C3–C2–C1	114.9(2)	O2–C1–C2	117.7(2)
C2–C1–O1	123.9(2)		
Dihedral angles [°]			
O1–C1–C2–C3	−174.7(2)	C1–C2–C3–N1	76.7(3)
O2–C1–C2–C3	4.9(3)		
Interatomic contacts [Å]			
N1–H3B···F4*i*	2.865(3)	O2–H2···F8	2.602(2)
O1–H1···F12*ii*	2.616(2)		

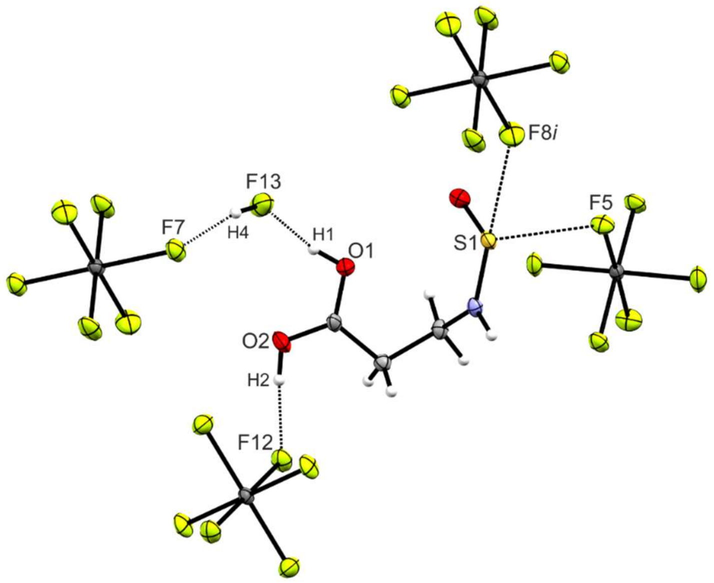

Figure S3. Projection of interatomic contacts in the $[C(OH)_2(CH_2)_2NHSO][SbF_6]_2 \cdot HF$ (**4**) crystal (50% probability displacement ellipsoids). Symmetry code: *i* = 1−x,1−y, 2−z.

Table S3. Selected bond lengths [Å] and angles [°] of $[C(OH)_2(CH_2)_2NHSO][SbF_6]_2 \cdot HF$ (**4**) with estimated standard deviations in parentheses. Symmetry codes: *i* = 1−x,1−y, 2−z.

Bond lengths [Å]			
N1–C3	1.488(7)	C1–O1	1.268(6)
C2–C3	1.513(6)	C1–O2	1.270(6)
C1–C2	1.489(8)	N1–S1	1.563(4)
S1–O3	1.431(4)		
Bond angles [°]			
N1–C3–C2	112.4(4)	O1–C1–O2	117.7(5)
C3–C2–C1	114.2(4)	O2–C1–C2	122.9(5)
C2–C1–O1	128.8(4)	C3–N1–S1	127.8(4)
N1–S1–O3	111.4(2)		
Dihedral angles [°]			
O1–C1–C2–C3	−5.6(7)	C1–C2–C3–N1	71.0(6)
O2–C1–C2–C3	175.2(5)	C2–C3–N1–S1	−115.7(5)
C3–N1–S1–O3	3.1(5)		
Interatomic contacts [Å]			
O2–H1···F12	2.626(4)	O1–H1···F13	2.544(6)
S1···F5	2.811(3)	S1···F8*i*	2.776(4)

Table S4. Experimental vibrational frequencies [cm^{-1}] of (**1**) and (**2**) and calculated vibrational frequencies [cm^{-1}] of $[C(O)F(CH_2)_2NH_3]^{+}\cdot HF$.

$[C(O)F(CH_2)_2NH_3][SbF_6]$ (1)		$[C(O)F(CH_2)_2NH_3][AsF_6]$ (2)		$[C(O)F(CH_2)_2NH_3]^{+}\cdot HF$	Assignment
IR	Raman	IR	Raman	Calc.[a] (IR/Raman)	
3283 (w)	3290 (8)	3303 (w, sh)	3302 (3)	3482 (122/56)	$\nu_{as}(NH_3)$
3244 (w)	3244 (15)	3238 (m)	3247 (9)	3355 (381/71)	$\nu_{as}(NH_3)$
3186 (vw)		3158 (w, sh)	3166 (4)	3242 (203/54)	$\nu_{s}(NH_3)$
3057 (vw)	3057 (26)	3066 (vw)	3067 (10)	3150 (0.6/45)	$\nu_{as}(CH_2)$
	3008 (26)		3028 (14)	3102 (3/91)	$\nu_{as}(CH_2)$
2976 (vw)	2976 (34)	2974 (vw)	2975 (16)	3093 (3/64)	$\nu_{s}(CH_2)$
2953 (vw)	2953 (61)	2951 (vw)	2951 (33)	3046 (3/111)	$\nu_{s}(CH_2)$
	2814 (8)		2795 (4)		?
			2757 (4)		?
	2704 (9)		2701 (3)		?
1812 (w)	1822 (28)	1814 (vw)	1826 (17)	1842 (228/13)	ν(CO)
1603 (w)	1601 (14)	1605 (vw)	1600 (10)	1687 (31/2)	$\delta_{as}(NH_3)$
1587 (w)	1584 (14)	1591 (vw)	1595 (11)	1656 (23/6)	$\delta_{as}(NH_3)$
1493 (w)		1483 (w)	1492 (6)	1509 (122/3)	$\delta_{s}(NH_3)$
	1475 (13)		1476 (12)	1505 (49/4)	$\delta(CH_2)$
1464 (vw)	1466 (20)			1451 (35/7)	$\delta(CH_2)$
1396 (w)	1406 (27)	1402 (vw)	1407 (17)	1427 (3/0.9)	$\omega(CH_2)$
1383 (w)	1385 (10)	1381 (vw)	1386 (5)	1409 (69/0.4)	$\omega(CH_2)$
1327 (w)	1328 (20)	1329 (vw)	1329 (11)	1354 (40/3)	τ(CH2)
1259 (w)	1258 (21)	1256 (vw)	1256 (13)	1284 (15/3)	$\tau(CH_2)$
			1204 (3)		?
1165 (w)	1157 (13)	1155 (w)	1157 (7)	1208 (174/2)	ν(CF)
1134 (w)	1135 (9)	1126 (vw)	1126 (4)	1145 (10/0.4)	$\rho(NH_3)$
1094 (w)	1091 (10)	1084 (w)	1084 (4)	1108 (94/0.7)	$\rho(NH_3)$
1045 (w)	1050 (21)	1041 (vw)	1043 (12)	1024 (9/2)	ν(CC)
943 (vw)	941 (11)	932 (vw)	938 (7)	959 (4/0.5)	$\rho(CH_2)$
912 (vw)	913 (13)	908 (w)	910 (7)	917 (37/1)	ν(CC)
854 (vw)	857 (33)	854 (w)	856 (23)	843 (2/8)	ν(CN)
806 (w)	812 (30)	806 (w)	810 (26)	819 (23/5)	$\rho(CH_2)$
				671 (13/1)	δ(CCO)
582 (s)	575 (24)	576 (s)	577 (16)	561 (7/1)	γ(COFC)
484 (m)	484 (29)	492 (s)	494 (14)	477 (4/2)	δ(CCF)
				353 (13/0.1)	$\tau(NH_3)$

				348 (19/0.9)	δ(CCN)
				262 (37/0.3)	δ(CCC)
				210 (39/0.3)	δ(CCN)$_{oop}$
				80 (0.4/0.1)	Skeletal vibration
693 (m)	679 (16)	702 (vs)	706 (8)		MF_6^-
659 (vs)	664 (27)	683 (vs, sh)	686 (100)		MF_6^-
646 (vs)	655 (100)	636 (s)	636 (7)		MF_6^-
559 (s)	649 (88)	550 (s)	551 (8)		MF_6^-
550 (s)	565 (27)		373 (44)		MF_6^-
	555 (15)		360 (12)		MF_6^-
	365 (24)		117 (15)		MF_6^-
	305 (21)				MF_6^-
	289 (26)				MF_6^-
	281 (67)				MF_6^-
	146 (22)				MF_6^-

[a] Calculated on the B3LYP/aug-cc-pVTZ level of theory. IR intensity in km/mol and Raman intensity in $Å^4$/u. Abbreviations for IR intensities: v = very, s = strong, m = medium, w = weak, br = broad. Experimental Raman activities are stated to a scale of 1 to 100. *M* = Sb, As.

Table S5. Experimental vibrational frequencies [cm^{-1}] of (**3**) and calculated vibrational frequencies [cm^{-1}] of $[C(OH)_2(CH_2)_2NH_3]^{2+}\cdot 3HF$.

**$[C(OH)_2(CH_2)_2NH_3][AsF_6]_2$ (3)**		**$[C(OH)_2(CH_2)_2NH_3]^{2+}\cdot 3HF$**	**Assignment**
**IR**	**Raman**	**Calc.[a] (IR/Raman)**	
3365 (m)	3268 (3)		?
3234 (vs)	3242 (6)	3467 (103/17)	$\nu_{as}(NH_3)$
	3198 (4)	3415 (86/48)	$\nu_{as}(NH_3)$
3159 (vs, sh)	3161 (3)	3206 (544/156)	$\nu_s(NH_3)$
3125 (s,sh)		3161 (1589/121)	ν(OH)
3054 (w, sh)	3053 (8)	3149 (5/33)	$\nu_{as}(CH_2)$
	3008 (10)	3099 (3/90)	$\nu_s(CH_2)$
2957 (vw)	2961 (11)	3070 (7/41)	$\nu_{as}(CH_2)$
		3050 (2141/122)	ν(OH)
2928 (vw)	2931 (14)	3021 (57/81)	$\nu_s(CH_2)$
	2801 (6)		?
	2701 (5)		?
1657 (m)	1656 (9)	1676 (19/4)	$\delta_{as}(NH_3)$
	1616 (9)	1664 (19/3)	$\delta_{as}(NH_3)$
1605 (w)		1653 (310/0.7)	ν(CO)
1589 (w)	1590 (11)	1568 (212/5)	ν(CO)
1506 (w)	1510 (8)	1552 (123/0.9)	$\delta_s(NH_3)$
1481 (m)	1476 (7)	1498 (12/4)	$\delta(CH_2)$
1466 (m)	1467 (16)	1440 (18/1)	$\omega(CH_2)$
1410 (w)	1408 (7)	1428 (27/5)	$\delta(CH_2)$
1366 (w)	1366 (24)	1397 (30/2)	$\omega(CH_2)$
1354 (w)	1357 (15)	1363 (27/3)	$\tau(CH_2)$
1312 (w)	1316 (8)	1329 (111/0.2)	δ(COH)
1292 (w)			?
1254 (vw)	1254 (11)	1292 (10/2)	$\tau(CH_2)$
1242 (vw)			?
1196 (w)	1214 (8)	1275 (214/5)	δ(COH)
1149 (vw)	1139 (8)	1151 (20/0.4)	$\rho(NH_3)$
1107 (w)	1113 (5)	1122 (27/0.3)	$\rho(NH_3)$
1043 (w)	1047 (14)	1032 (11/3)	ν(CC)
1022 (w)			?
974 (w)		962 (54/0.2)	$\delta(COH)_{oop}$
939 (w)	943 (11)	947 (17/0.6)	$\tau(CH_2)$
906 (w)	922 (10)	915 (2/3)	ν(CC)

		888 (128/0.2)	δ$(COH)_{oop}$
856 (m)	858 (10)	841 (11/3)	ν(CN)
814 (s)	817 (26)	806 (13/9)	δ(OCO)
619 (s)	627 (8)	660 (4/0.7)	δ(OCC)
538 (m)	544 (8)	573 (11/2)	γ(CCO_2)
490 (s)	497 (13)	496 (16/2)	δ(OCC)
		361 (29/0.8)	δ(CCN)
		324 (19/0.3)	τ(NH_3)
	257 (7)	287 (51/0.5)	δ(CCC)
	127 (13)	98 (8/0.8)	Skeletal vibrations
		52 (8/0.2)	Skeletal vibrations
723 (vs)	717 (41)		AsF_6^-
712 (vs)	683 (100)		AsF_6^-
967 (vs)	588 (18)		AsF_6^-
675 (vs)	576 (11)		AsF_6^-
580 (s)	423 (10)		AsF_6^-
563 (s)	371 (45)		AsF_6^-
424 (m)			AsF_6^-

[a] Calculated on the B3LYP/aug-cc-pVTZ level of theory. IR intensity in km/mol and Raman intensity in $Å^4$/u. Abbreviations for IR intensities: v = very, s = strong, m = medium, w = weak, br = broad. Experimental Raman activities are stated to a scale of 1 to 100.

Table S6. Selected NBOs (BD = 2-center bond; LP = 1-center valence lone pair; BD* = 2-center antibond) of *β*-propiolactam together with calculated values for occupancy and s- and p-character.[a]

Bond	Occupancy	Energy	s-, p- character
BD(1) C3-C2	1.97 e⁻	−0.58681	C3 s (26.00%), p 2.84 (73.86%)
			C2 s (24.66%), p 3.05 (75.14%)
BD(1) C3-N1	1.99 e⁻	−0.71947	C3 s (22.01%), p 3.54 (77.84%)
			N1 s (31.58%), p 2.16 (68.25%)
BD(1) C2-C1	1.98 e⁻	−0.59149	C2 s (23.57%), p 3.24 (76.28%)
			C1 s (33.02%), p 2.02 (66.84%)
BD(1) C1-N1	1.99 e⁻	−0.80129	C1 s (30.68%), p 2.25 (69.17%)
			N1 s (35.18%), p 1.84 (64.62%)
BD*(1) C1-N1	0.08 e⁻	0.43573	C1 s (30.68%), p 2.25 (69.17%)
			N1 s (35.18%), p 1.84 (64.62%)
BD(1) C1-O1	2.00 e⁻	−1.08942	C1 s (36.42%), p 1.74 (63.45%)
			O1 s (41.04%), p 1.42 (58.28%)
BD*(1) C1-O1	0.02 e⁻	0.64457	C1 s (36.42%), p 1.74 (63.45%)
			O1 s (41.04%), p 1.42 (58.28%)
BD(2) C1-O1	2.00 e⁻	−0.37404	C1 s (0.00%), p 1.00 (99.76%)
			O1 s (0.00%), p 1.00 (99.57%)
BD*(2) C1-O1	0.28 e⁻	0.01880	C1 s (0.00%), p 1.00 (99.76%)
			O1 s (0.00%), p 1.00 (99.57%)
LP(1) N1	1.70 e⁻	−0.26373	N1 s (0.00%), p 1.00 (99.94%)
LP(1) O1	1.98 e⁻	−0.69300	O1 s (58.86%), p 0.70 (41.00%)
LP(2) O1	1.78 e⁻	−0.25031	O1 s (0.01%), p 99.99 (99.65%)

[a] Calculated on the B3LYP/aug-cc-pVTZ level of theory.

Table S7. Selected NBOs (BD = 2-center bond; LP = 1-center valence lone pair; BD* = 2-center antibond) of the *N*-protonated species of *β*-propiolactam together with calculated values for occupancy and s- and p-character.[a]

Bond	Occupancy	Energy	s-, p-character
BD(1) C3-C2	1.98 e^-	−0.82163	C3 s (27.43%), p 2.64 (72.43%)
			C2 s (25.85%), p 2.86 (73.93%)
BD(1) C3-N1	1.99 e^-	−0.93087	C3 s (19.52%), p 4.12 (80.33%)
			N1 s (28.01%), p 2.56 (71.84%)
BD(1) C2-C1	1.98 e^-	−0.88147	C2 s (22.65%), p 3.41 (77.16%)
			C1 s (43.93%), p 1.27 (55.98%)
BD(1) C1-N1	1.99 e^-	−0.82358	C1 s (19.16%), p 4.21 (80.66%)
			N1 s (20.21%), p 3.94 (79.72%)
BD*(1) C1-N1	0.26 e^-	−0.12976	C1 s (19.16%), p 4.21 (80.66%)
			N1 s (20.21%), p 3.94 (79.72%)
BD(1) C1-O1	2.00 e^-	−1.43141	C1 s (37.26%), p 1.68 (62.62%)
			O1 s (45.05%), p 1.20 (54.19%)
BD*(1) C1-O1	0.02 e^-	0.47551	C1 s (37.26%), p 1.68 (62.62%)
			O1 s (45.05%), p 1.20 (54.19%)
BD(2) C1-O1	1.99 e^-	−0.64604	C1 s (0.00%), p 1.00 (99.77%)
			O1 s (0.00%), p 1.00 (99.43%)
BD*(2) C1-O1	0.07 e^-	−0.20634	C1 s (0.00%), p 1.00 (99.77%)
			O1 s (0.00%), p 1.00 (99.43%)
LP(1) O1	1.98 e^-	−0.93211	O1 s (54.73%), p 0.82 (45.07%)
LP(2) O1	1.70 e^-	−0.50655	O1 s (0.13%), p 99.99 (99.38%)

[a] Calculated on the B3LYP/aug-cc-pVTZ level of theory.

Table S8. Selected NBOs (BD = 2-center bond; LP = 1-center valence lone pair; BD* = 2-center antibond) of the *O*-protonated species of *β*-propiolactam together with calculated values for occupancy and s- and p-character.[a]

Bond	Occupancy	Energy	s-, p-character
BD(1) C3-C2	1.97 e^{-}	−0.77803	C3 s (25.89%), p 2.86 (73.94%)
			C2 s (23.94%), p 3.17 (75.81%)
BD(1) C3-N1	1.98 e^{-}	−0.91098	C3 s (19.45%), p 4.13 (80.38%)
			N1 s (30.65%), p 2.26 (69.19%)
BD(1) C2-C1	1.97 e^{-}	−0.84572	C2 s (22.95%), p 3.35 (76.82%)
			C1 s (35.84%), p 1.79 (64.04%)
BD(1) C1-N1	2.00 e^{-}	−1.10674	C1 s (33.01%), p 2.03 (66.89%)
			N1 s (35.52%), p 1.81 (64.17%)
BD*(1)C1-N1	0.03 e^{-}	0.28658	C1 s (33.01%), p 2.03 (66.89%)
			N1 s (35.52%), p 1.81 (64.17%)
BD(2) C1-N1	1.97 e^{-}	−0.59417	C1 s (0.00%), p 1.00 (99.65%)
			N1 s (0.00%), p 1.00 (99.82%)
BD*(2) C1-N1	0.31 e^{-}	−0.25520	C1 s (0.00%), p 1.00 (99.65%)
			N1 s (0.00%), p 1.00 (99.82%)
BD(1) C1-O1	2.00 e^{-}	−1.25812	C1 s (31.23%), p 2.20 (68.63%)
			O1 s (36.36%), p 1.74 (63.19%)
BD*(1) C1-O1	0.04 e^{-}	0.20163	C1 s (31.23%), p 2.20 (68.63%)
			O1 s (36.36%), p 1.74 (63.19%)
LP(1) O1	1.96 e^{-}	−0.83045	O1 s (41.54%), p 1.40 (58.25%)
LP(2) O1	1.76 e^{-}	−0.56886	O1 s (0.00%), p 1.00 (99.62%)

[a] Calculated on the B3LYP/aug-cc-pVTZ level of theory.

Table S9. Comparison of selected NBOs together with electron occupancies (occ.) and s- and p-character of *β*-propiolactam, the *N*-protonated species and the *O*-protonated species of *β*-propiolactam.[a]

Bond	***β*-propiolactam**		***N*-protonated species of *β*-propiolactam**		***O*-protonated species of *β*-propiolactam**	
	**Occ.**	**s- and p-character**	**Occ.**	**s- and p-character**	**Occ.**	**s- and p-character**
C1–O1 (σ)	2.00	C1 s (36.42%), p 1.74 (63.45%)	2.00	C1 s (37.26%), p 1.68 (62.62%)	2.00	C1 s (31.23%), p 2.20 (68.63%)
		O1 s (41.04%), p 1.42 (58.28%)		O1 s (45.05%), p 1.20 (54.19%)		O1 s (36.36%), p 1.74 (63.19%)
C1–O1 (σ*)	0.02	C1 s (36.42%), p 1.74 (63.45%)	0.02	C1 s (37.26%), p 1.68 (62.62%)	0.04	C1 s (31.23%), p 2.20 (68.63%)
		O1 s (41.04%), p 1.42 (58.28%)		O1 s (45.05%), p 1.20 (54.19%)		O1 s (36.36%), p 1.74 (63.19%)
C1–O1 (π)	2.00	C1 s (0.00%), p 1.00 (99.76%)	1.99	C1 s (0.00%), p 1.00 (99.77%)		
		O1 s (0.00%), p 1.00 (99.57%)		O1 s (0.00%), p 1.00 (99.43%)		
C1–O1 (π*)	0.28	C1 s (0.00%), p 1.00 (99.76%)	0.07	C1 s (0.00%), p 1.00 (99.77%)		
		O1 s (0.00%), p 1.00 (99.57%)		O1 s (0.00%), p 1.00 (99.43%)		
C1–N1 (σ)	1.99	C1 s (30.68%), p 2.25 (69.17%)	1.99	C1 s (19.16%), p 4.21 (80.66%)	2.00	C1 s (33.01%), p 2.03 (66.89%)
		N1 s (35.18%), p 1.84 (64.62%)		N1 s (20.21%), p 3.94 (79.72%)		N1 s (35.52%), p 1.81 (64.17%)
C1–N1 (σ*)	0.08	C1 s (30.68%), p 2.25 (69.17%)	0.26	C1 s (19.16%), p 4.21 (80.66%)	0.03	C1 s (33.01%), p 2.03 (66.89%)
		N1 s (35.18%), p 1.84 (64.62%)		N1 s (20.21%), p 3.94 (79.72%)		N1 s (35.52%), p 1.81 (64.17%)
C1–N1 (π)					1.97	C1 s (0.00%), p 1.00 (99.65%)
						N1 s (0.00%), p 1.00 (99.82%)
C1–N1 (π*)					0.31	C1 s (0.00%), p 1.00 (99.65%)
						N1 s (0.00%), p 1.00 (99.82%)

[a]Calculated on the B3LYP/aug-cc-pVTZ level of theory.

Table S10. Crystal data and structure refinement for $[C(O)F(CH_2)_2NH_3][SbF_6]$ (**1**), $[C(OH)_2(CH_2)_2NH_3][AsF_6]_2$ (**3**) and $[C(OH)_2(CH_2)_2NHSO][SbF_6]_2 \cdot HF$ (**4**).

	$[C(O)F(CH_2)_2NH_3][SbF_6]$ (**1**)	$[C(OH)_2(CH_2)_2NH_3][AsF_6]_2$ (**3**)	$[C(OH)_2(CH_2)_2NHSO][SbF_6]_2 \cdot HF$ (**4**)
Empirical formula	C3 H7 F7 N O Sb	C3 H9 F12 N O2 As2	C3 H8 F13 N O3 S Sb2
M_r	327.85	468.95	628.66
Crystal system	triclinic	monoclinic	triclinic
Space group	$P\overline{1}$	$P2_1/n$	$P\overline{1}$
a [Å]	5.4383(4)	8.1022(2)	7.8845(8)
b [Å]	7.4689(6)	11.6917(4)	8.6948(9)
c [Å]	10.7243(13)	12.7565(5)	12.0433(7)
α [°]	100.215(9)	90	83.250(7)
β [°]	103.362(9)	92.465(3)	73.866(8)
γ [°]	90.033(7)	90	69.580(9)
V [Å^3]	416.70(7)	1207.29(7)	743.07(13)
Z	2	4	2
ρ_{calcd} [gcm^{-3}]	2.613	2.580	2.810
μ[mm^{-1}]	3.394	5.693	3.929
$\lambda_{Mo K\alpha}$	0.71073	0.71073	0.71073
F(000)	308	896	584
T[K]	160(2)	126(2)	116(2)
hkl range	–7:6; –9:9;–12:14	–9:11; –16:16;–18:16	−9:10; −11:11;−16:15
refl. measured	3716	12429	6425
refl. unique	2063	3684	3686
R_{int}	0.0336	0.0379	0.0428
parameters	130	199	240
R(F)/*w*R(F^2)$^{a)}$	0.0332/ 0.0634	0.0426/ 0.0703	0.0462/0.0879
weighting scheme$^{b)}$	0.0245	0.0293	0.0303
S(GoF)$^{c)}$	1.032	1.041	1.047
residual density [eÅ^{-3}]	0.857/ −0.848	0.806/ –0.677	1.950/ −1.308
device type	Oxford XCalibur	Oxford XCalibur	Oxford XCalibur
solution/refinement	SHELXT	SHELXT	SHELXT
CCDC	2062961	2062962	2062963

a) $R_1 = \Sigma||F_o|-|F_c||/\Sigma|F_o|$; b) $wR_2 = [\Sigma[w(F_o^2-F_c^2)^2]/\Sigma[w(F_o)^2]]^{1/2}$; $w = [\sigma_c^2(F_o^2)+(xP)^2+yP]^{-1}$; $P = (F_o^2+2F_c^2)/3$ c) GoF $= \{\Sigma[w(F_o^2-F_c^2)^2]/(n-p)\}^{1/2}$ (*n* = number of reflexions; *p* = total number of parameters).

Table S11. Cartesian coordinates of calculated minimum structure of $[C(O)F(CH_2)_2NH_3]^{+}\cdot HF$ at the B3LYP/aug-cc-pVTZ level of theory.

Atom	x	y	z
F	4.603415	1.424747	4.811568
O	5.102853	0.394984	6.687191
N	3.106168	-0.074080	8.484731
C	2.385879	-0.321565	7.177724
H	1.318152	-0.287769	7.374816
H	2.656037	-1.327666	6.865649
C	2.780524	0.705409	6.121039
H	2.310227	0.441803	5.173530
H	2.415365	1.706041	6.369740
C	4.271656	0.797185	5.930506
H	2.808161	0.807548	8.919090
H	2.942973	-0.830739	9.148538
H	4.115599	-0.015276	8.270154
F	2.042938	2.481602	9.178240
H	1.905854	3.182375	9.775085

Table S12. Cartesian coordinates of calculated minimum structure of $[C(OH)_2(CH_2)_2NH_3]^{2+}\cdot 3HF$ at the B3LYP/aug-cc-pVTZ level of theory.

Atom	x	y	z
O	1.484897	5.474129	4.855161
O	3.000457	4.017730	5.466283
N	2.016381	1.405456	6.721071
C	1.288623	1.773755	5.447332
H	0.448802	1.087901	5.367172
H	1.973277	1.565340	4.628788
C	1.778577	4.296413	5.218152
C	0.760550	3.208926	5.399267
H	0.051557	3.274591	4.569861
H	0.173017	3.464182	6.289507
H	0.523761	5.666139	4.650520
H	3.645717	4.782190	5.336524
H	2.863632	1.961860	6.854449
H	1.429488	1.516010	7.551769
H	2.299261	0.408772	6.675438
F	-1.010999	5.869421	4.342791
H	-1.471643	6.626226	4.040522
F	4.568103	6.042419	5.205280
H	5.475217	6.203312	5.047001
F	2.581653	-1.225070	6.145449
H	2.848845	-2.093173	6.360860

Table S13. Cartesian coordinates of calculated minimum structure of the transition state and concurrently starting point for IRC calculations at the MP2/aug-cc-pVTZ level of theory.

Atom	x	y	z
C	-0.700793	1.069375	-0.156676
C	-1.299493	-0.303755	0.332876
H	-1.093296	1.315808	-1.141393
H	-0.675249	1.930613	0.502090
H	-1.355470	-0.411146	1.410180
N	-0.052788	-1.054960	-0.198715
H	1.233521	-1.039531	0.353584
C	0.532974	0.251275	-0.211879
O	1.668355	0.191597	0.272686
H	-2.200209	-0.621683	-0.178213
H	-0.202634	-1.538784	-1.085637

Table S14. Cartesian coordinates of calculated minimum structure of endpoint of IRC calculation (*O*-protonation) on MP2/aug-cc-pVTZ level of theory.

**Atom**	**x**	**y**	**z**
C	0.549342	1.070483	0.000089
C	1.514323	-0.150697	-0.000098
H	0.567269	1.688305	0.894278
H	0.567197	1.688667	-0.893842
H	2.102227	-0.286047	-0.901350
N	0.284797	-0.996837	0.000008
H	-2.283922	-0.693155	0.000003
C	-0.521115	0.032779	0.000015
O	-1.803382	0.153561	0.000022
H	2.102477	-0.286146	0.900974
H	0.127230	-1.999155	0.000033

Table S15. Cartesian coordinates of calculated minimum structure of endpoint of IRC calculation (*N*-protonation) on MP2/aug-cc-pVTZ level of theory.

**Atom**	**x**	**y**	**z**
C	-0.709294	0.996584	0.018536
C	-1.484711	-0.330860	0.077228
H	-0.883785	1.618029	-0.859315
H	-0.759210	1.622485	0.909110
H	-2.008566	-0.517729	1.007554
N	-0.230310	-1.173454	-0.006555
H	-0.021944	-1.744909	0.814004
C	0.604948	0.270831	-0.071491
O	1.758972	0.392680	-0.153904
H	-2.131501	-0.524372	-0.770761
H	-0.137535	-1.752049	-0.843167

Table S16. Cartesian coordinates of calculated minimum structure of *β*-propiolactam on the B3LYP/aug-cc-pVTZ level of theory.

**Atom**	**x**	**y**	**z**
C	-0.656253	-0.845432	0.000427
C	0.894473	-0.768356	0.000038
H	-1.098157	-1.291023	-0.890421
H	-1.096967	-1.287204	0.893870
H	1.385374	-1.154041	-0.890139
H	1.385086	-1.153129	0.890788
C	0.706632	0.765037	-0.000714
N	-0.657427	0.622852	-0.002377
H	-1.399099	1.305676	0.000673
O	1.428260	1.726693	-0.000054

Table S17. Cartesian coordinates of calculated minimum structure of the *N*-protonated species of *β*-propiolactam on the B3LYP/aug-cc-pVTZ level of theory.

**Atom**	**x**	**y**	**z**
C	0.344468	1.144977	-0.000093
C	1.446234	0.066240	0.000035
H	0.292441	1.783374	0.882969
H	0.292253	1.783723	-0.882872
H	2.065343	0.034417	-0.889476
N	0.444484	-1.078323	-0.000032
H	0.447384	-1.673981	-0.828378
C	-0.739445	0.104531	0.000019
O	-1.878624	-0.079239	0.000048
H	2.064851	0.034665	0.889907
H	0.447798	-1.674517	0.827933

Table S18. Cartesian coordinates of calculated minimum structure of the *O*-protonated species of *β*-propiolactam on the B3LYP/aug-cc-pVTZ level of theory.

Atom	x	y	z
C	1.518485	-0.153337	0.000295
C	0.549257	1.070691	-0.000098
H	2.111846	-0.288340	-0.898342
H	2.111017	-0.288224	0.899416
H	0.567204	1.694345	-0.891356
H	0.567091	1.694772	0.891027
C	-0.523415	0.030312	-0.000247
O	-1.803928	0.145416	0.000032
N	0.283116	-0.996307	-0.000286
H	0.128080	-1.997811	-0.000145
H	-2.301585	-0.689921	0.001445
